# Effect of lycopene supplementation on cardiovascular parameters and markers of inflammation and oxidation in patients with coronary vascular disease

**DOI:** 10.1002/fsn3.734

**Published:** 2018-08-13

**Authors:** Ivan M. Petyaev, Pavel Y. Dovgalevsky, Victor A. Klochkov, Natalya E. Chalyk, Dmitry V. Pristensky, Marina P. Chernyshova, Ruzan Udumyan, Taron Kocharyan, Nigel H. Kyle, Marina V. Lozbiakova, Yuriy K. Bashmakov

**Affiliations:** ^1^ Lycotec Ltd Cambridge UK; ^2^ Institute of Cardiology Saratov Russia; ^3^ Orebro University Hospital and School of Health and Medical Sciences Orebro University Örebro Sweden

**Keywords:** cardiovascular disease, formulation, inflammation, lycopene, lycosome

## Abstract

Oxidative stress and antioxidant deficiency play a pivotal role in initiation, development, and outcomes of cardiovascular disease. Pharmacokinetic parameters as well as the impact of highly bioavailable lycopene on cardiovascular variables, markers of inflammation and oxidation were investigated during a 30‐day clinical trial in patients with coronary vascular disease. The patients were randomized into two major groups and were supplemented with a single 7 mg daily dose of lycopene ingested either in the form of lactolycopene (68 patients) or in the form of lycosome‐formulated GA lycopene (74 patients). The endpoints included cardiovascular function parameters, serum lipids, and four markers of oxidative stress and inflammation. Ingestion of lycosome‐formulated lycopene increased serum lycopene levels by 2.9‐ and 4.3‐fold, respectively, after 2 and 4 weeks of the trial, whereas supplementation with lactolycopene upregulated serum lycopene by half‐fold only after 4 weeks of ingestion. Lycosome formulation of lycopene resulted by the end of the trial in a threefold reduction in *Chlamydia pneumoniae* IgG and reduction to the same degree of the inflammatory oxidative damage marker. The decrease in oxidized LDL caused by lycosome‐formulated lycopene was fivefold. Moreover, supplementation with lycosome‐formulated lycopene was accompanied by a significant increase in tissue oxygenation and flow‐mediated dilation by the end of the observational period. In contrast, lactolycopene did not cause any significant changes in the parameters studied. Therefore, enhanced bioavailability of lycopene promotes its antioxidant and anti‐inflammatory functions and endorses a positive effect of lycopene on cardiovascular system.

## 1 INTRODUCTION

Lycopene is a polyunsaturated multihydrocarbon phytochemical from the tetraterpene carotenoid family with strong antioxidant properties. It occurs naturally in a variety of fruits and vegetables including tomatoes, watermelon, and papaya (Jain, Mehra, & Swarnakar, 2015). Mammalian species cannot synthesize lycopene and are wholly dependent on ingestion of lycopene from dietary sources. The 13 linearly aligned double bonds in the lycopene molecule make it the most powerful antiradical compound in the carotenoid family and epitomize its biological function (Igielska‐Kalwat, Gościańska, & Nowak, 2015). There is a growing body of scientific evidence that the antioxidant properties of lycopene confer resistance to cardiovascular disease, cancer, diabetes, and inflammatory diseases (Bahonar, Saadatnia, Khorvash, Maracy, & Khosravi, 2017; Gajowik & Dobrzyńska, 2014; Petyaev, 2016; Roohbakhsh, Karimi, & Iranshahi, 2017). Lycopene is classed as a nonessential nutrient and therefore, there is no recommended daily intake. Average intake of lycopene is very low even in the developed world and reflects mostly the amount ingested with cooked tomato products. Daily intake of lycopene varies from 0.7 mg (Finland) to 1.3 mg (Germany), while a much higher range (3.7–16.1 mg) is reported for the United States (Porrini & Riso, 2005). It has been revealed recently that the participation of lycopene in biological oxidation reactions and scavenging of free radicals leads to irreversible degradation of the lycopene with the formation of end products excreted from the human body (Muller et al., 2011; Petyaev, 2016). Thus, aging and diseases accompanied by oxidative stress (atherosclerosis, diabetes, and cancer) might be accompanied by lycopene depletion and development of lycopene deficiency. However, even well‐designed nutritional strategies to replenish lycopene levels in human cells and tissues might be not adequate enough to manage lycopene deficiency due to its poor intestinal absorption, low bioavailability, and reduced ability of the liver to incorporate lycopene into lipoprotein carriers (Borel, Desmarchelier, Nowicki, & Bott, 2015; Maru, Hudlikar, Kumar, Gandhi, & Mahimkar, 2016; Muller et al., 2011; Petyaev, 2016). Therefore, the development of new nutraceutical formulations of lycopene with enhanced bioavailability and which address impaired processing in the liver has become an important task for modern nutritional science in order to create effective lycopene supplementation.

In the present work, we investigated pharmacokinetic parameters as well as the impact of highly bioavailable lycopene on cardiovascular variables, markers of inflammation, and oxidation in patients with coronary vascular disease.

## 2 MATERIALS AND METHODS

### 2.1 Study design

The study was conducted at the Institute of Cardiology, the Ministry of Health of the Russian Federation (Saratov, Russian Federation) by Lycotec Ltd (Cambridge, United Kingdom). The protocol was approved by the local ethics committee. All patients were informed of the purpose and goals of the study and had signed a consent form before enrollment and participation in the study.

### 2.2 Inclusion/exclusion criteria

Coronary vascular disease (CVD) was defined and diagnosed according to the American College of Cardiology and American Heart Association Guidelines (Smith et al., 2006). CVD patients eligible for the study were screened for levels of blood markers for oxidative stress and inflammation. One hundred and fifty‐five CVD patients were positive for both types of markers and were randomized into two groups for the study. Thirteen patients were unable to complete the study for reasons not related to the intake of the test products.

Eligibility of patients for the study was determined by the following inclusion/exclusion criteria.

Among *Inclusion Criteria* were as follows: Caucasian male or female subjects 45–73 years old, ability to sign an informed consent, light‐to‐moderate smokers (≤10 cigarettes daily), elevated markers for inflammation (*Chl.pn*‐IgG ELISA × 10^3^ ≥ 400 and CRP ≥ 6 μg/ml), elevated serum markers for oxidative stress (LDL‐Px ELISA × 10^3^ ≥ 200 U/ml and IOD ≥ 40 μM/ml), no participation in other dietary trials during the last 3 months before enrollment and duration of study, willingness and ability to comply with the study protocol for the duration of the study.

*Exclusion criteria* were as follows: Unwillingness to sign informed consent, unable to comply with the protocol for the duration of the study, history of myocardial infarction in the 3 months preceding the study, ejection fraction (EF) < 45%, significant medical condition that would impact safety considerations (e.g., significantly elevated LFT, hepatitis, severe dermatitis, uncontrolled diabetes, cancer, severe GI disease, fibromyalgia, renal failure, recent CVA (cerebrovascular accident), pancreatitis, respiratory diseases, epilepsy, etc.), compulsive alcohol abuse (>10 drinks weekly), or regular exposure to other substances of abuse, participation in other nutritional or pharmaceutical studies, resting heart rate of >100 beats per minute or <50 beats per minute, positive test for tuberculosis, HIV, or hepatitis B, unable to tolerate phlebotomy, special diets in the 4 weeks prior to the study (e.g., liquid, protein, raw food diet), tomato intolerance.

### 2.3 Products

Two lycopene products were studied. The first was a lycopene formulation developed by Nestle Inc (Lacto‐Lycopene, LL, Switzerland). The second was microencapsulated GA Lycopene developed by Lycotec Ltd. (Cambridge, UK). The daily dose for both formulations was one capsule of 7 mg of lycopene. All products were to be taken with the main evening meal. The period of administration was 1 month.

## 3 METHODS

### 3.1 BMI, pulse rate, and BP

Measurements of body mass index, BMI, body mass of the patients, and their height were carried out in the morning and BMI was calculated in kg/m^2^. Pulse rate, systolic and diastolic blood pressure, SBP and DBP, were recorded three times on the left arm of the seated patient after 15 min of rest. The time between measurements was greater than 2 min. The mean result for each parameter was calculated. All body and vascular parameters were recorded in the morning between 8 and 10 a.m.

### 3.2 Flow‐Mediated Dilation (FMD)

Endothelium‐dependent flow‐mediated vasodilatation was measured in accordance with widely accepted guidelines (Corretti et al., 2002). Patients were screened under ambient conditions at the same time of the morning in a supine position. High‐resolution ultrasound was applied at the same anatomical landmark of a section of the brachial artery for a period of 30 s before and during the peak of reactive hyperemia. It was positioned prior to sphygmomanometer cuff occlusion and 1 min after its deflation. The level of inflation was 50 mm Hg above the patient's systolic blood pressure and it continued for 5 min. Arterial diameter was imaged above the antecubital fossa in a longitudinal scan by duplex ultrasound with linear phase‐array transducer. FMD was calculated as a change in poststimulus diameter as a percentage of the baseline diameter (Bianchini, Faita, Gemignani, Giannoni, & Demi, 2006).

### 3.3 Ankle‐Brachial Index (ABI)

ABI was measured between left and right brachial arteries, the one with the highest SBP was chosen, and between left and right tibial arteries, the one with the highest SBP was also chosen for the assessment of ABI. For this purpose, a continuous‐wave Doppler probe was used after patients had been in a supine position for at least 15 min of rest (Anderson, 2006).

### 3.4 Tissue oxygenation

Thenar eminence and forearm muscles of the patients were used as a tissue target for the assessment of oxygen saturation, StO_2_, or combined level of oxygenated hemoglobin and myoglobin. StO_2_ was assessed by continuous wavelength near‐infrared spectroscopy, NIRS, with wide‐gap second‐derivative (In Spectra, Hutchinson Technology, MN, USA). The measurements were taken at different time points. The recording was initiated after 15 min of rest in a supine position before occlusion of the brachial artery. It was then continued during stagnant ischemia induced by rapidly inflating the cuff to 50 mm Hg above systolic BP. The ischemia lasted for 3 min, and the recording period lasted for another 5 min after that until StO_2_ was stabilized (Bezemer et al., 2009; Gómez et al., 2009).

The area under the hyperemic curve, AUC, of the recorded signal for the settling time in the postocclusion period was then calculated as described earlier in % O_2_/min.

### 3.5 Blood collection

Blood was collected in the morning from the arm veins of patients following night fast. The serum was separated from the rest of the clotted mass by centrifugation, and aliquots were then stored at −80°C prior to analysis.

### 3.6 Biochemistry and inflammatory markers

Glucose, total cholesterol, TC, triglycerides, TG, high‐density cholesterol, HDL, low‐density cholesterol, LDL, C‐reactive protein, CRP and Chlamydia pneumoniae IgG (*Chl.pn*‐IgG) were determined using commercially available analytical kits according to the manufacturers’ instructions (ByoSystems, Medac, R&D Systems).

### 3.7 Lycopene quantitative analysis

The lycopene concentration in all serum samples was measured in duplicate by high‐performance liquid chromatography (Diwadkar‐Navsariwala et al., 2003) with modifications. Briefly, 400 μl of serum was mixed with 400 μl of ethanol and was extracted twice with 2 ml hexane. The combined hexane layers were evaporated to dryness in a vacuum (Scan Speed 32 centrifuge) and the residue reconstituted to a volume of 100 μl in sample solution (absolute ethanol—methylene chloride, 5:1, v/v). The specimens were centrifuged again (15 min at 10,000 *g*) and clear supernatant was transferred to HPLC vials. Five microliters of the extract was injected into an Acquity HSS T3 75 x 2.1 mm 1.8 μm column (Waters, MA, USA) preceded by a Acquity HSS T3 1.8 μm VanGuard precolumn (Waters) and eluted isocratically at 45^°^C with the mobile phase (acetonitrile—0.08% phosphoric acid solution—tert‐Butyl methyl ether, 70:5:25, v/v/v) at a flow rate of 0.5 ml/min. The lycopene peak was detected by a photodiode array detector (Waters) at 474 nm. The peak area was measured using Empower 3 software (Waters). The lycopene concentration in serum samples was calculated by reference to an analytical standard (lycopene from tomato, L9879; Sigma, USA).

### 3.8 Inflammatory Oxidative Damage (IOD)

Serum samples were incubated overnight in 0.05 M PBS acetate buffer (pH 5.6) to imitate the type of oxidative damage which occurs during the release of lysosomes following neutrophil degranulation. The following morning, the reaction was stopped using trichloroacetic acid. The concentration of the end products such as malonic dialdehyde (MDA), and other possible thiobarbituric acid‐reactive substances (TBARS), was then measured by colorimetry (Yagi, 1978) using reagents and kits from Cayman Chemical (MC, USA).

### 3.9 LDL‐Px

Activity of serum LDL peroxidase proteins, which include IgG with superoxide dismutase activity, was measured as described previously (Petyaev, Mitchinson, Hunt, & Coussons, 1998).

### 3.10 Statistics

For the assessment of normally distributed parameters, the Shapiro–Wilk method was used. Student's  *t* test was then applied for both paired and unpaired samples. In cases where parameters were not normally distributed, the Mann–Whitney  *U*  test and Kruskal–Wallis test were used. ANOVA and ANCOVA were used with post hoc analysis (Statistica 9 suite, StatSoft; Inc.). Statistical significance between two‐tailed parameters was considered to be *p* < 0.05.

## 4 RESULTS

### 4.1 Clinical groups and randomization

As can be seen from Table 1, there was no significant difference in age, gender, body mass index, fasting glucose, and lipids (total cholesterol, triglycerides, LDL, HDL) between the two clinical groups of the study. The patients belonging to the groups also had similar parameters of cardiovascular health suggesting an altogether successful and valid randomization. No significant differences were found for parameters of lipid peroxidation in the groups (results not shown). All patients remained normoglycemic from enrollment to the endpoint of the study.

**Table 1:** Group characterizations

Baseline characteristics of the enrolled volunteers (Mean ± *SD*)		
Variable	Lactolycopene	Lycosome GA lycopene
Number of Patients	68	74
Males	35	40
Females	33	34
Age	56.1 ± 5.9	54.2 ± 5.3
Light/Moderate smokers		
Body mass index	28.5 ± 3.2	26.4 ± 3.9
Fasting glucose mg/dl	91.0 ± 9.9	85.1 ± 8.2
Total cholesterol mg/dl	191.0 ± 12.6	184.5 ± 14.1
Triglycerides mg/dl	133.4 ± 13.9	128.0 ± 12.7
LDL mg/dl	140.6 ± 11.2	147.9 ± 13.1
HDL mg/dl	40.2 ± 3.3	39.5 ± 3.1
Pulse rate per min	70.1 ± 4.0	75.6 ± 5.2
ABI	1.12 ± 0.05	0.99 ± 0.07
FMD	12.0 ± 0.59	11.2 ± 0.39
Blood pressure		
Systolic	121.5 ± 7.9	120.8 ± 6.4
Diastolic	75.7 ± 5.2	75.1 ± 6.0
StO_2_	13.0 ± 0.6	12.0 ± 0.5

Note

Patients were screened, enrolled, and randomized into two major groups of the study. Baseline characteristics were measured at “0” time point of the study as described in the “Materials and Methods” section.

### 4.2 Pharmacokinetic parameters

Lycopene measurements did not show any significant differences between the two groups of the study in the pretreatment period. Ingestion of lactolycopene did not affect serum lycopene level after 2 weeks of consumption (median value of 58.0 ng/mg; 5/95% CI: 69.2/48.4; vs. pretreatment value of 58.0 ng/mg; 5/95% CI: 76.4/55.0, *p* > 0.05). However, there was some increase in serum lycopene content by the end of the observational period (up to median value of 87.0 ng/mg; 5/95% CI: 93.9/72.2, *p* < 0.05).

**Figure 1:**
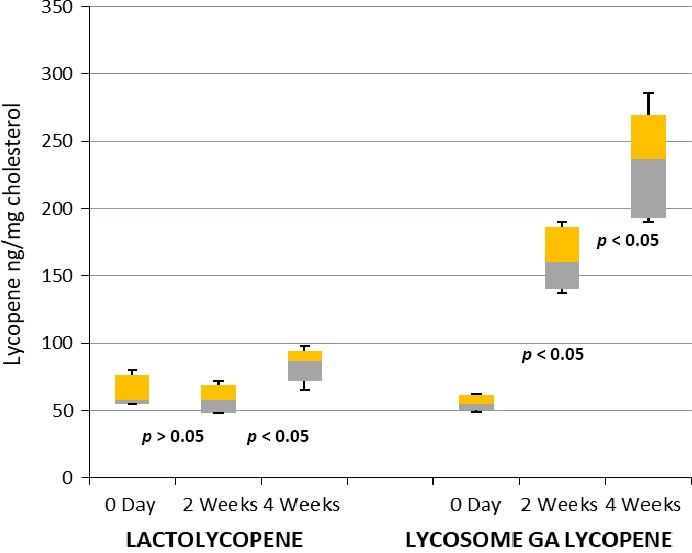
Box and whisker analysis of serum lycopene levels. Patients were screened, enrolled, and randomized into two major groups of the study. Serum lycopene concentration was measured after 2 and 4 weeks following daily ingestion of 7 mg of lactolycopene or 7 mg of lycosome GA lycopene as described in the “Materials and Methods” section

In contrast, the lycosome formulation of GA lycopene markedly increased serum lycopene concentration with an increase in median over control level (55.0 ng/mg) up to 160.0 ng/mg and 237.0 ng/mg (*p* = 0.0001) after 2 and 4 weeks of treatment, respectively (Figure 1).

### 4.3 Lipid profile

**Figure 2:**
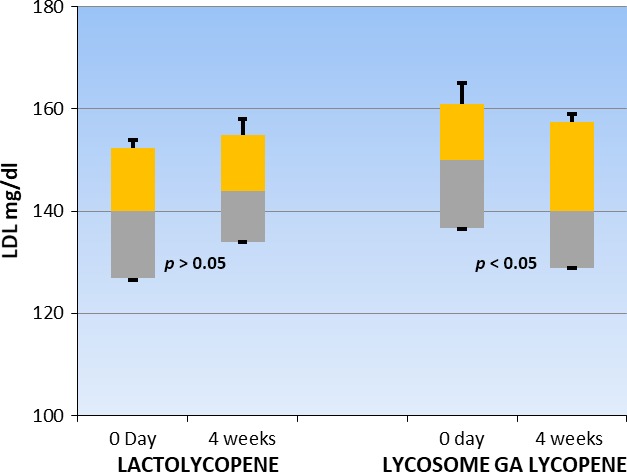
Box and whisker analysis of serum ldl level. Patients were screened, enrolled, and randomized into two major groups of the study. Serum LDL were measured after 4 weeks following daily ingestion of 7 mg of lactolycopene or 7 mg of lycosome GA lycopene as described in the “Materials and Methods” section

Supplementation with lactolycopene and GA lycopene did not significantly affect parameters of lipid profile in patients belonging to either group of the study (Table 2). However, detailed analysis of variant distribution showed (Figure 2) that ingestion of lycosome‐formulated lycopene caused a tendency toward serum LDL reduction at the endpoint of the study with borderline level of statistical significance. Such an effect was not seen in the patients treated with lactolycopene.

**Table 2:** Changes in lipid profile

Serum lipids mg/dl (Medians with 5/95% CIs)		
Variable	Lactolycopene	Lycosome GA lycopene
Total cholesterol		
Day 0	189.0 (193.6/180.8)	184.0 (190/0/176.2)
4 weeks	190 (195.0/182.0)*	183.0 (190.6/171.6)*
Triglycerides		
Day 0	135.0 (145.85/128.2)	143.0 (158.2/132.4)
4 weeks	133.0 (148.9/125.8)*	137.0 (147.9/120.8)*
LDL		
Day 0	140.0 (152.6/125.8)	150.0 (161.5/136.6)
4 weeks	144.0 (155.2/134.0)*	140.0 (156.2/128.8) **
HDL		
Day 0	41.0 (44.6/39.0)	41.0 (44.0/39.0)
4 weeks	41.0 (42.6/39.4)*	42.0 (43.0/39.4)*

Notes

Patients were screened, enrolled, and randomized into two major groups of the study. Serum lipids were measured after 4 weeks following daily ingestion of 7 mg of lactolycopene or 7 mg of lycosome GA lycopene as described in the “Materials and Methods” section.

*p >* *0.05; **p <* *0.05.

### 4.4 Cardiovascular parameters

Neither lycopene formulation changed pulse rate or parameters of systemic blood pressure in patients by the endpoint of the study (Table 3). Similarly, no changes in Ankle–Brachial Index Test were observed in either group of the study. However, there were statistically significant increases in values for FMD (increase in median by 1.1 points or increase over baseline by 10.7%) and StO_2_ (increase in median by 1.9 points, or increase over baseline by 15.9%) by the end of the observational period in patients treated with the lycosome formulation of lycopene. Interestingly, such an increase did not take place when the lactolycopene formulation was ingested.

**Table 3:** Changes in cardiovascular functions

Cardiovascular parameters (Medians with 5/95%% CIs)		
Variable	Lactolycopene	Lycosome GA lycopene
Pulse rate		
Day 0	70.0 (76.9/65.0)	75.0 (84.5/68.4)
4 weeks	69.0 (76.3/63.2)*	74.0 (84.7/68.8)*
Blood pressure		
Systolic		
Day 0	120 (132.6/110.4)	122.0 (130.0/110.8)
4 weeks	125.0 (131.2/117.4)*	119.0 (128.9/118)*
Diastolic		
0	73.0 (86.3/67.8)	75.0 (84.9/67)
4 weeks	73.0 (85.0/67.0)*	79 (86.2/69.0)*
ABI		
Day 0	1.1 (1.2/1.04)	0.95 (1.1/0.92)
4 weeks	1.1 (1.16/1.10)*	0.93 (0.94/0.90)*
FMD		
Day 0	11.2 (11.7/10.3)	10.3 (10.8/9.68)
4 weeks	11.1 (11.5/10.6)*	11.4 (12.3/11.1)**
StO_2_		
Day 0	12.6 (13.7/11.7)	11.9 (11.9/10.3)
4 weeks	12.9 (13.3/11.8)*	13.8 (14.7/11.3)**

Note

Patients were screened, enrolled, and randomized into two major groups of the study. Cardiovascular parameters were assessed after 4 weeks following daily ingestion of 7 mg of lactolycopene or 7 mg of lycosome GA lycopene as described in the “Materials and Methods” section.

### 4.5 Parameters of inflammation and oxidative stress

**Figure 3:**
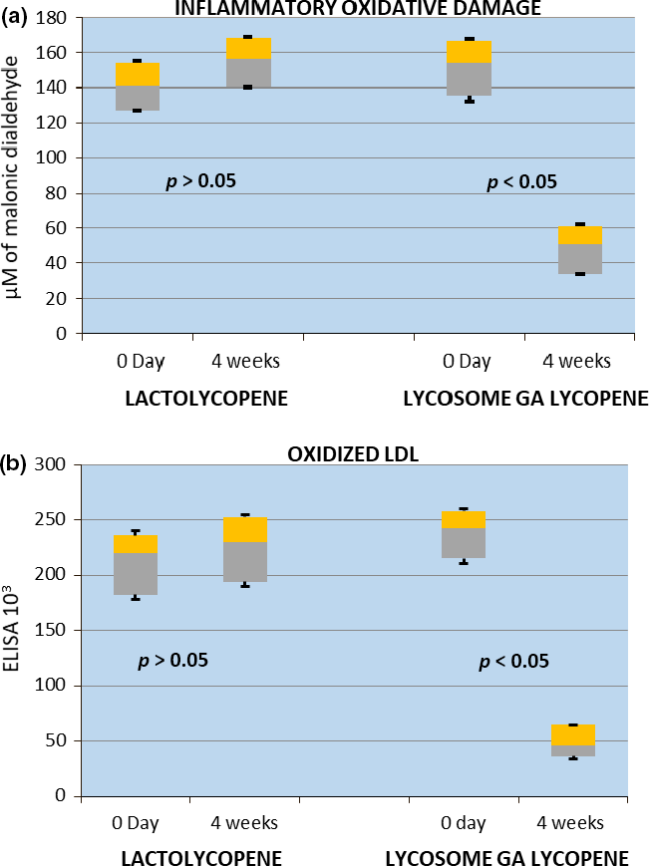
Box and whisker analysis of inflammatory oxidative damage (a) and oxidized LDL (b) values. Patients were screened, enrolled, and randomized into two major groups of the study. Inflammatory oxidative damage and oxidized LDL values were assessed after 4 weeks following daily ingestion of 7 mg of lactolycopene or 7 mg of lycosome GA lycopene as described in the “Materials and Methods” section

Lycopene supplementation had no impact on serum CRP level (Table 4). However, there was a differential pattern of regulation of other inflammatory parameters between the two formulations of lycopene (Table 4 and Figure 3). lactolycopene did not affect inflammatory markers by the end of the interventional period, whereas lycosome‐formulated lycopene significantly reduced antichlamydial IgG levels (threefold reduction, *p* < 0.05), concentration of oxidized LDL (fivefold decrease, *p* < 0.05) and IOD value (threefold reduction, *p* < 0.05) as compared to pretreatment values.

**Table 4:** Changes of inflammation and oxidation markers

Inflammation and oxidation markers (Medians with 5/95%% CIs)		
Variable	Lactolycopene	Lycosome galycopene
*C. pneumoniae* IgG		
Day 0	433.0 (468.3/391.0)	432.0 (444.6/390.5)
4 weeks	488.0 (506.9/436.6)*	144.0 (164.2/122.2)**
C‐reactive protein		
Day 0	6.0 (7.0/5.26)	6.8 (7.4/5.6)
4 weeks	6.2 (6.8/5.2)*	6.1 (7.3/5.4)*
Oxidized LDL		
Day 0	220.0 (236.5/182.4)	243.0 (254.7/215.4)
4 weeks	240.0 (253.2/194)*	46.0 (65.0/36.4)**
Inflammatory oxidative		
Damage		
Day 0	141.0 (153.9/127.0)	154.0 (167.2/135.6)
4 weeks	156.0 (168.2/140.0)*	51.0 (61.5/34.0)**

Note

Patients were screened, enrolled, and randomized into two major groups of the study. Markers of inflammation and oxidation were assessed after 4 weeks following daily ingestion of 7 mg of lactolycopene or 7 mg of lycosome GA lycopene as described in the “Materials and Methods” section.

## 5 DISCUSSION

There are a number of recently published clinical trials and meta‐analysis data revealing beneficial health effects of lycopene (Cicero & Colletti, 2015; Mahmoodnia, Mohammadi, & Masumi, 2017; Singh & Bagewadi, 2017; van Die et al., 2016). However, only few of them (Graff et al., 2017; Zhu, Gertz, Cai, & Burri, 2016) adequately address the issue of lycopene bioavailability in volunteers. As we shown above, the ingestion of lycopene does not necessarily translate into sustainable increase in circulating lycopene levels and rather reflects the type of nutraceutical formulation of lycopene used for supplementation. In particular, ingestion of lycosome‐formulated lycopene leads to a significant increase in serum lycopene levels, exceeding the baseline values by 2.9 and 4.3 times, respectively, after 2 and 4 weeks of administration, whereas supplementation with lactolycopene was significantly less effective in upregulating of serum lycopene levels in cardiovascular patients. Improved lycopene bioavailability is most likely to explain a prominent reduction in oxidized LDL level and values of inflammatory oxidative damage (IOD) seen in the enrolled patients treated with lycosome formulation of GA lycopene. It is also important that most prominent reduction in oxidized LDL and IOD values took place at end the endpoint of observational period, when serum lycopene concentration reached a highest level. Moreover, supplementation with lycosome‐formulated lycopene was accompanied by measurable increases in values of FMD and StO_2_. These observations are in a good agreement with recently published (Cheng et al., 2017) systematic review and meta‐analysis data summarizing effects of lycopene and tomato supplementation on cardiovascular health.

Moreover, decline in oxidized LDL and IOD values caused by GA lycopene supplementation was accompanied by a threefold reduction in *Chlamydia pneumoniae* IgG which is known to be an important marker of oxidative stress and inflammation in cardiovascular patients (Di Pietro, Filardo, Falasca, Turriziani, & Sessa, 2017). This observation is in a good agreement with our recently published results (Zigangirova et al., 2017) revealing a strong inhibitory effect of lycopene on *C. trachomatis* and *C. pneumoniae* infections in cultured cells. However, under the conditions used in our study, we did not observe any significant changes in the markers of oxidation and inflammation or in cardiovascular parameters for patients supplemented with lactolycopene, although there was a half‐fold increase in serum lycopene level in patients with cardiovascular disease after 1 month of supplementation. These data suggest the mild increase in circulating lycopene level is not sufficient enough to have an impact on the state of biological oxidation and cardiovascular function.

It has to be mentioned that lycopene has very low bioavailability rate. In raw fruit, lycopene is localized in chloroplasts, a barely digestible organelle of plant cells (Petyaev, 2016). Thermal food processing, especially in the presence of cooking oils, promotes micellization of lycopene and increases its intestinal absorption rate in a multifold manner (Dhuique‐Mayer et al., 2016). To be transported in the body, lycopene must be incorporated into lipoprotein molecules. This process takes place in hepatocytes and intestinal epithelial cells and can be severely impaired with age, metabolic syndrome, and atherosclerosis (Borel et al., 2015; Diwadkar‐Navsariwala et al., 2003; Petyaev, 2016; Roohbakhsh et al., 2017). As result of this, even lycopene which has been absorbed cannot be incorporated into its lipoprotein carriers and people develop not a nutritional but a functional deficiency. To overcome this problem, the GA lycopene formulation contains a phospholipid chaperone molecule, which can serve as scaffolding and facilitate lipoprotein–lycopene coassembly. As we have reported above, administration of lycosome GA lycopene formulation resulted in a significant increase in serum lycopene concentration in patients with cardiovascular disease. Lycosome formulation of lycopene containing a phospholipid chaperone may provide a significant level of protection for “cargo” lycopene molecules from stomach acidity and digestive enzymes. In our previous work, we demonstrated that this protection resulted in an enhanced bioavailability rate and efficacy for a number of nutraceutical molecules such as resveratrol, whey protein peptides, cocoa flavanols, and certain pharmaceuticals (Bashmakov et al., 2014; Petyaev, 2015; Petyaev, Dovgalevsky, Chalyk, Klochkov, & Kyle, 2014; Petyaev, Dovgalevsky, Klochkov, Chalyk, & Kyle, 2012).

Oxidative stress plays a pivotal role in the initiation, development, and outcomes of cardiovascular disease (He & Zuo, 2015). As we shown above, reduction in the markers of biological oxidation (oxidized LDL, IOD) in cardiovascular patients treated with GA lycopene translates into increase in tissue oxygenation and flow‐mediated dilation at the end of the observational period. Both parameters are important systemic measures of cardiovascular health (Di Minno et al., 2016; Schröder, 2016) which suggests that antioxidant supplementation may have a measurable impact on the outcomes of cardiovascular disease.

Finally, our study has some limitations. It remains to be established in future if enhanced bioavailability lycopene can retain its antioxidant effect on lipoprotein oxidation and cardiovascular parameters in the long term. Studies with longer duration of lycopene supplementation and placebo control are required. It would also be important to assess how this nutraceutical intervention may affect other clinical forms of cardiovascular and cerebrovascular pathologies. The impact of different lycopene isomers on oxidative and cardiovascular parameters should also be studied. Nevertheless, our results suggest that lycopene supplementation holds significant promise in the management of cardiovascular disease and might be used for the correction of inflammatory status and cardiovascular parameters in CVD patients.

## CONFLICT OF INTERESTS

The authors declare no conflict of interest involved.

## ETHICAL STATEMENTS

The study was approved by the Institutional Review Boards of the Saratov's Institute of Cardiology (Russian Federation) and Lycotec Ltd (UK). Written informed consent was obtained from all study participants. The study was conducted in accordance with the Declaration of Helsinki and the Guidelines accepted by USA/European agencies for clinical studies as well as current regulatory acts for medical research in the Russian Federation.
